# Inhibition modulated by self-efficacy: An event-related potential study

**DOI:** 10.3389/fpsyg.2022.904132

**Published:** 2022-09-27

**Authors:** Hong Shi

**Affiliations:** Department of English Language and Literature, School of Foreign Languages, China University of Petroleum, Beijing, China

**Keywords:** inhibition, self-efficacy, Go/No-Go task, neural correlate, ERP

## Abstract

Inhibition, associated with self-efficacy, enables people to control thought and action and inhibit disturbing stimulus and impulsion and has certain evolutionary significance. This study analyzed the neural correlates of inhibition modulated by self-efficacy. Self-efficacy was assessed by using the survey adapted from the Motivated Strategies for Learning Questionnaire. Fifty college students divided into low and high self-efficacy groups participated in the experiments. Their ability to conduct inhibitory control was studied through Go/No-Go tasks. During the tasks, we recorded students’ brain activity, focusing on N2 and P3 components in the event-related potential (ERP). Larger No-Go N2 amplitudes for the high self-efficacy group were found compared with the low self-efficacy group. Conflict detection as represented by N2 was modulated by self-efficacy, whereas conflict inhibition as represented by P3 was not modulated by self-efficacy. The highly self-efficacious students were more capable of detecting conflicts but not necessarily more capable of inhibiting action given that conflict was detected. Taken together, these findings offer neurophysiological evidence of the important regulatory role of self-efficacy in inhibitory control ability development.

## Introduction

Inhibition or inhibitory control, referred to as response inhibition, is an executive function defined as the ability to deliberately withhold or override a dominant, prepotent (habitual), or automatic response to resist distraction or temptation and to achieve the desired goal (Nigg, 2000; Gagne, 2017; Kloo and Sodian, 2017). Inhibition in response to a stimulus is associated with self-efficacy (Bembenutty, 2011; Mcauley et al., 2011). Self-efficacy refers to self-perceptions or beliefs of the capability to learn or perform tasks at designated levels (Bandura, 1997). Students with high self-efficacy may have stronger inhibitory control ability and would continue working even when a task-irrelevant temptation to stop calls for attention, and they may be better able to exit from an ongoing action sequence in response to a task-relevant signal to do so. However, students with low self-efficacy beliefs may have weaker inhibitory control ability and tend to succumb to temptation and let disruptive thoughts interfere with performance or miss a task-relevant signal to interrupt an ongoing action (Bembenutty, 2011; Mcauley et al., 2011; Wang, 2018). Previous studies have documented individuals’ behavioral performance on measures of inhibition, and for example, there is evidence that inhibitory control is related to academic skills (e.g., Cragg and Nation, 2008; Allan et al., 2014; Dekker et al., 2017; Morgan et al., 2019; Litkowski et al., 2020). Furthermore, previous studies identified a modulation role for self-efficacy in inhibition (e.g., Mcauley et al., 2011; Liew, 2012; Gärtner et al., 2018), but few studies have examined neural underpinnings of inhibition modulated by self-efficacy. The present study investigates the underlying brain neural correlates of inhibition modulated by self-efficacy using the event-related potential (ERP) method. ERPs can provide evidence of the brain mechanisms of response inhibition, and the ERP literature has examined N2 and P3 components to analyze the inhibition process (e.g., Smith et al., 2006; Enriquez-Geppert et al., 2014; Wang, 2018). Therefore, we want to clarify the potential mechanisms underlying inhibition modulated by self-efficacy through the analysis of N2 and P3 components.

The common way to examine the neural correlates of inhibition is using Go/No-Go tasks (Rueda et al., 2005; Wiebe et al., 2012; Smith et al., 2013). In a typical Go/No-Go task, individuals respond to a frequently occurring stimulus type (Go trials), and inhibit their response when a less frequently occurring stimulus is presented (No-Go trials). The extent to which participants are able to withhold a response on No-Go trials serves as a measure of their inhibitory control abilities. The bulk of the Go/No-Go ERP studies focusing on inhibition have examined the No-Go N2-P3 complex (e.g., Lahat et al., 2010; Hoyniak, 2017). Some current views claimed that the conflict detection operation, which is a part of the inhibition process, is associated with the N2, whereas inhibition of the action operation is associated with the P3 (Smith et al., 2008; Albert et al., 2013; Enriquez-Geppert et al., 2014). The N2, an increased frontal scalp negativity, is a wave within 150–400 ms after the onset of the stimulus, which peaks at approximately 300 ms post-stimulus onset. The N2 component can be used to index conflict detection between Go and No-Go response tendencies, and its amplitude is largest when the response conflict is high. Randall and Smith (2011) called it the conflict detection hypothesis of the N2. In many experiments, the N2 components are greater in the No-Go trials compared with the Go trials (e.g., Jodo and Kayama, 1992; Hoyniak, 2017; Lahat et al., 2010; Waldvogel et al., 2000). Studies revealed that the N2 was the unlikely equivalent of proper motor inhibition, and the N2 was evoked when stimulus constellations were associated with conflicts in information processing even though a response has to be executed (Huster et al., 2013). Enriquez-Geppert et al. (2010) suggested an association of the N2 with conflict-related effects with less frequently occurring trial types. Frequent responses are prepotent, eventually leading to conflicts at the response representation level when infrequent responses have to be made (Braver et al., 2001; Jones et al., 2002). Regarding N2, although almost no research examined the neurocognitive correlates of inhibition modulated by self-efficacy, researchers have investigated inhibition regulated by emotional induction or anxiety. A person’s affective states and actions are closely associated with self-efficacy (Bandura, 1997), and they could result in improved inhibitory control and increases in associated aspects of brain activity (Farbiash and Berger, 2015). Students with increased attention and higher effortful brain activation tended to view the execution of tasks as under their control. For example, Farbiash and Berger (2015) reported inhibition of No-Go trials was associated with larger N2 amplitudes during negative emotional induction for children aged 5–6. Hum et al. (2013) also found significantly larger No-Go N2 amplitudes for 8–12 year olds with anxiety than those without anxiety. The other manifestation of the inhibition mechanism is P3. It is the positive component that appears in the frontal center. Its manifestation is roughly a positive wave in the 300–600 ms range. The P3 amplitude differences have been found in response to Go versus No-Go trials (Ramautar et al., 2004; Dimoska et al., 2006). The P3 amplitude is larger in the No-Go trials (e.g., Bokura et al., 2001; Ciesielski et al., 2004). It seems that the recent predominant literature on Go/No-Go tasks identified that the P3 was directly related to the suppression of overt motor response (Huster et al., 2013). The majority of analyses indicate that P3 originates from multiple brain regions including frontal and temporo-parietal areas (Polich, 2007). Wang (2018) examined the brain inhibitory effect of self-efficacy of college students to English biological and non-biological vocabulary stimuli and identified that the No-Go P3 amplitude in the high self-efficacy group was larger than that in the low self-efficacy group, and indicated that students with high self-efficacy had better inhibitory control ability. Rosen (2010) adopted a different task—a flanker task (a task that varies task difficulty without changing the nature of the task due to its use of congruent and incongruent flanking stimuli, and the incongruent task requires greater interference control to inhibit task-irrelevant stimuli and execute the correct response) and found self-efficacy was related to enhanced stimulus processing, as evidenced by larger P3 amplitudes. Themanson and Rosen (2014) also found a positive relationship between self-efficacy and P3 amplitude during the completion of a flanker task. But these studies represented specific research designs and did not provide enough evidence for inhibition mechanisms modulated by self-efficacy. Furthermore, although some related studies adopted Go/No-Go tasks, both No-Go N2 and No-Go P3 are not consistently found associated with self-efficacy. The present study thus intends to use Go/No-Go tasks to clarify the inhibition process modulated by self-efficacy through the analysis of N2 and P3 components so that we can learn more about people’s control over thought and action to allow them to reduce interference and maintain goal-oriented actions. The experimental hypothesis is that participants with high levels of self-efficacy have stronger inhibitory control ability on stimulus and interference, and thus have larger No-Go N2 and No-Go P3 in terms of test indicators.

## Materials and methods

The ability to conduct inhibitory control is studied through Go/No-Go tasks. The experimental indicators are No-Go N2 and No-Go P3.

### Participants

This study was conducted at a public university of science and engineering in the city of Beijing, China. We used convenience sampling for this study. There were 61 students who had previously been tested for self-efficacy and appropriate samples were selected based on their pretest scores. Twenty-four college students with high self-efficacy were selected, and 26 college students with low self-efficacy were selected. There were 12 females and 12 males, 11 undergraduate students and 13 graduate students in the high self-efficacy group; and 14 females and 12 males, 12 undergraduate students and 14 graduate students in the low self-efficacy group. The experimental participants were right-handed, with normal or corrected vision, and they had no history of mental illness. At the request of the Academic Ethics Committee of the university, they signed informed consent to participate in this study. Table 1 (see Appendix B) shows the demographic information of the participants.

### Self-reported instrument

Self-efficacy was assessed before Go/No-Go tasks by using the survey adapted from the Motivated Strategies for Learning Questionnaire (MSLQ) (Pintrich et al., 1993). The MSLQ has been validated and used in many studies (e.g., Pintrich et al., 1991, 1993; Pintrich, 2003). This questionnaire is a self-report instrument designed to assess college students’ motivational orientations and self-regulated learning, and the self-efficacy subscale in MSLQ is designed particularly to measure the self-efficacy beliefs of students (Pintrich et al., 1991). This study mainly used the self-efficacy subscale in MSLQ to measure the self-efficacy of participants (see Appendix A). The value of Cronbach’s alpha for the self-efficacy scale was 0.902. Students rated themselves on a 9-point Likert scale, from 1 (not at all true of me) to 9 (very true of me). It was a median split (the score for high self-efficacy is ≥ 5, and for low self-efficacy is < 5). The mean score for the high self-efficacy group is 7.06 (range of scores is 5.38–8.79), and the mean score for the low self-efficacy group is 3.58 (range of scores 2.31–4.83).

### Stimuli and procedure

Before the experiment, the experimental procedure was described and the students participated in the experiment in a relaxed state. Pictures including single triangle and double triangle were selected as the stimulus. The participants were instructed to respond by pressing the button “/” (right hand) or “z” (left hand) as quickly as they could whenever the “Go” stimulus (double triangle) was presented and not to press the button when the “No-Go” stimulus (single triangle) was presented. A fixation cross to orient attention to the middle of the screen was presented for 500 ms. Stimuli were presented for 50 ms, and participants could respond anytime within the onset of the stimulus and the interstimulus interval (950 ms). A practice phase of 20 trials with feedback was given. Each condition consisted of 150 test trials. Go stimuli were presented for most of the trials (80%). All experimental tasks were presented using the E-prime software 3.0.

### Electroencephalogram recording and preprocessing

Electroencephalogram (EEG) signals were continuously recorded with the NeuroLab digital amplifier system (Yiran Sunny Technology Co., Ltd., Beijing, China), using NeuCap with Ag/AgCl electrodes at 32 sites according to the extended international 10–20 system. The reference electrode was placed on the nose tip. The nose-tip reference was converted into bilateral mastoid for reference in offline data analysis. Vertical and horizontal electrooculography (EOG) signals were recorded with two electrodes placed above and below the right eye and with two electrodes at the right and left outer canthi of the eyes, respectively. Data were recorded continuously at a sampling rate of 1,000 Hz and filtered offline with a bandpass of 0.01–100 Hz. Electrode impedance was maintained below 5 kΩ throughout the experiment.

The EEGLab software^1^ was used to analyze EEG data. Blinks were corrected using an ICA procedure. The plotted average, condition-specific activation had to account for the N2/P3 time course. The EOG components were identified and selected according to the topographical maps that had to show a fronto-central scalp distribution, which is usually seen with the N2 and P3. One of the EOG components was removed on average. They were performed on all subjects. Remaining artifacts exceeding ± 100μV in amplitude or containing a change of over 100μV within a period of 50 ms were rejected. After performing EOG correction and visual inspection, only artifact-free trials were considered (rejected epochs, 3%).

The EEG was segmented in epochs of 700 ms, time-locked to picture onset, and included a 100 ms pre-stimulus baseline. Trials contaminated by amplifier clipping, bursts of EMG activity, or peak-to-peak deflection exceeding ± 100 μV were excluded from averaging (excluded 5%). The averaged ERP waveforms were low-pass filtered at 30 Hz. Filter frequencies correspond to the half-amplitude cut-off (24 dB/octave rolloff).

### Data analysis

For response times, only Go trials with correct responses were included. There were no response times for correct response No-Go trials, as no response was made if participants inhibited the response successfully. Means for all the conditions are presented in Table 2. Since the accuracy is high, the ERP analyses are restricted to correct-response trials.

The ERP literature has examined Fz, Cz, and Pz to analyze the inhibition process or self-efficacy effects (e.g., Themanson et al., 2008; Zhao et al., 2019). The Fz, Cz, and Pz electrodes in the electroencephalogram record were selected for the statistical analysis of mean amplitude over the time window of 200–400 ms and 400–600 ms. Repeated-measures ANOVA was conducted with Go/No-Go trials and electrode sites (Fz, Cz, and Pz) as within-subject factors and self-efficacy level (high vs. low) as between-subject factors. The Greenhouse-Geisser correction was applied where sphericity was violated. When a main effect or interaction was significant, *post hoc* comparisons were performed with Bonferroni correction. Analyses were conducted using the SPSS software (Version 26, SPSS Inc.).

## Results

### N2 amplitude

While repeated-measures ANOVA (see Table 3 in Appendix B) revealed the main effect of Go/No-Go trials (*F*(1,48) = 4.48, *p* = 0.04, partial η^2^ = 0.09) and the main effect of the site (*F*(2,96) = 6.21, *p* = 0.003, partial η^2^ = 0.11), with the Go trials (−2.59 μV) exhibiting smaller amplitude than the No-Go trials (−2.83 μV), and the N2 amplitude at the Cz (- 3.45 μV) significantly larger than those at the Fz (- 1.19 μV) and Pz (- 2.77 μV), there was no interaction among self-efficacy level, Go/No-Go trials, and electrode sites, between Go/No-Go trials and electrode sites, and between self-efficacy and electrode sites. But the main effect of self-efficacy was significant (*F*(1,48) = 7.12, *p* = 0.01, partial η^2^ = 0.13). There is interaction between self-efficacy and Go/No-Go trials (*F*(1,48) = 4.76, *p* = 0.03, partial η^2^ = 0.09). The amplitudes of No-Go N2 were significantly larger in the high self-efficacy group (- 3.42 μV) than in the low self-efficacy group (- 0.83 μV, *p* < 0.03).

### P3 amplitude

Repeated-measures ANOVA (see Table 4 in Appendix B) revealed no main effect of self-efficacy and Go/No-Go trials, but there was a main effect of the site (*F*(2,96) = 6.74, *p* = 0.002, partial η^2^ = 0.12), with the P3 amplitude at the Pz (0.76 μV) significantly larger than those at the Cz (- 0.47 μV) and Fz (- 0.70 μV). A significant interaction effect was found between Go/No-Go trials and electrode sites (*F*(2,96) = 18.77, *p* < 0.001, partial η^2^ = 0.28), with no other interaction effect observed. When we explored each site, self-efficacy level and Go/No-Go trials significantly interacted at only Pz (*F*(1,48) = 5.02, *p* = 0.01, partial η^2^ = 0.12), and an enhanced P3 amplitude on No-Go trials in high self-efficacy group (- 0.30 μV) compared with low self-efficacy group (- 0.61 μV) was identified, but this difference was not significant (*p* > 0.1).

**FIGURE 1:**
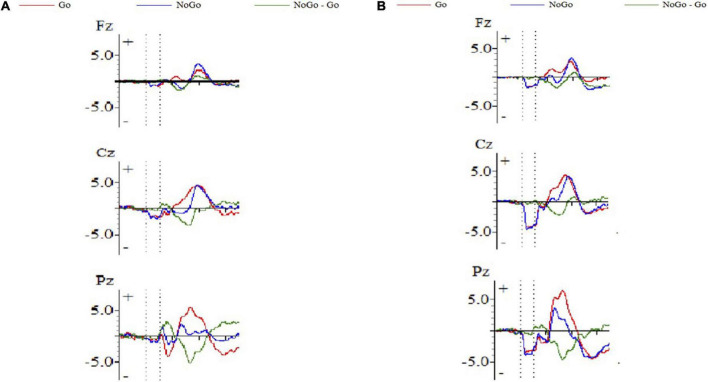
The ERP waveform diagram of Fz, Cz, and Pz. **(A)** High self-efficacy group; **(B)** low self-efficacy group. The red line represents the waveform result of the Go trials, and the blue line represents the waveform result of the No-Go trials.

**FIGURE 2:**
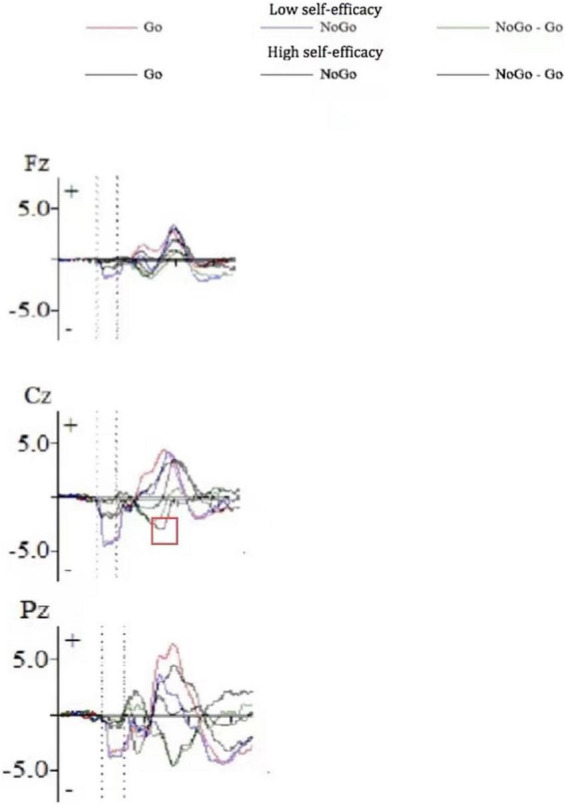
The ERP waveform diagram of Fz, Cz, and Pz for the difference between high self-efficacy group and low self-efficacy group.

**FIGURE 3:**
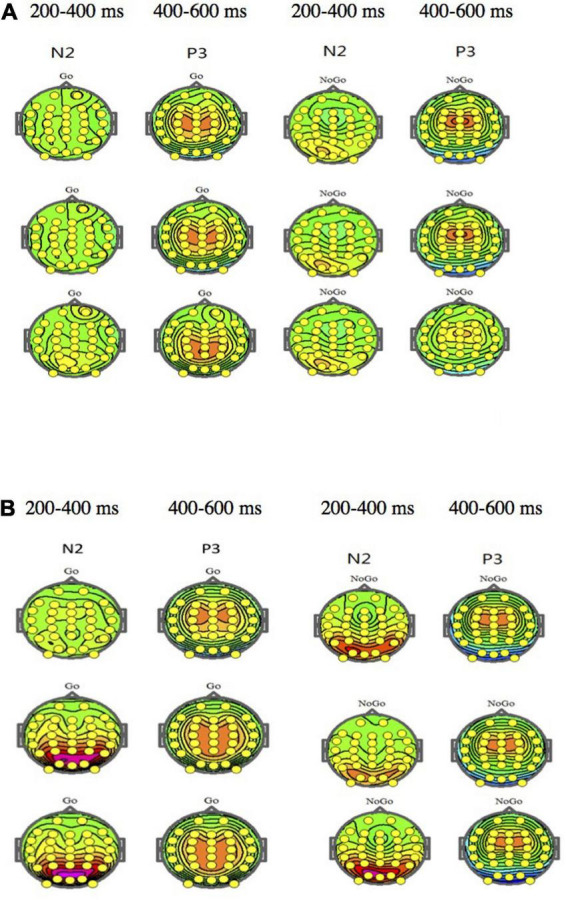
**(A)** The topographical map of the high self-efficacy group. **(B)** The topographical map of the low self-efficacy group.

Figure 1 shows the ERP waveform of electrode sites Fz, Cz, and Pz of high self-efficacy group (Figure 1A) and low self-efficacy group (Figure 1B). We can see that the overall wave amplitude development trend of the N2 and P3 waveforms was obvious. In different task conditions, the amplitudes of No-Go N2 and No-Go P3 were generally larger than that of Go N2 and Go P3 respectively. Figure 2 shows that compared with the low self-efficacy group the amplitudes of No-Go N2 in the high self-efficacy group were generally larger than that of Go N2, especially at the site Cz. Figure 3 shows the topographical map of the high self-efficacy group (Figure 3A) and the low self-efficacy group (Figure 3B). From the location of the brain region, the maximum amplitude of P3 occurred in the frontal center area.

## Discussions

This study investigated the neural correlates of inhibition modulated by self-efficacy. Two groups (low self-efficacy vs. high self-efficacy) of 50 college students participated in the experiments. We adopted Go/No-Go tasks and selected two ERP components associated with inhibitions—N2 and P3. The amplitudes of No-Go N2 were significantly larger in the high self-efficacy group compared with the low self-efficacy group. But there was no significant difference in P3 amplitudes on No-Go trials between the high self-efficacy group and the low self-efficacy group.

The finding of larger No-Go N2 amplitudes in the high self-efficacy group indicates that a person with a higher self-efficacy level is more likely to have a stronger ability to detect a conflict. According to Randall and Smith (2011)’s conflict detection hypothesis of the N2, it was used as a neural index of the ability to detect conflict between Go and No-Go response tendencies. Students with higher self-efficacy proved more sensitive to stimuli that presented a temptation to respond in accord with the prepotent action in an ongoing series of actions but in fact required a different response. Thus, it appears that higher self-efficacious students tend to regulate their behaviors in response to conflicts or challenges by “amping up” cortical activities that have become more efficient with the level of self-efficacy. According to Bandura (1977, 1986), high self-efficacy improves cognitive performance since it enhances on-task attention. This increased attention leads to greater focusing, which improves the ability to detect and monitor conflicts or irrelevant stimuli (Farbiash and Berger, 2015). High self-efficacy also enhances motivational aspects (Bandura, 1977, 1986), which facilitates conflict monitoring during a Go/No-Go task (Leue et al., 2012). A person’s affective states and actions are closely associated with self-efficacy (Bandura, 1997). The finding of the present study is, to some extent, in line with a study conducted by Lewis et al. (2006) that found the N2 on No-Go trials was greater during conditions of negative emotion induction. They explained that children’s performance was better when they were emotionally distressed, and the larger N2 amplitudes reflected higher effortful brain activation during the negative emotional experience (Lewis and Stieben, 2004). Negative emotion can result in improved inhibitory control and increases in associated aspects of brain activity (Farbiash and Berger, 2015). It seems that negative emotion, inhibitory control, and the greater N2 on No-Go trials might have some chain effects. The larger N2 amplitudes reflected higher effortful brain activation of students with high self-efficacy, which is also supported by Caraway et al. (2003) and they stated, “self-efficacy determines the aspect of task engagement including which tasks individuals choose to take on, the amount effort, persistence, and perseverance they demonstrate with regard to the task, and their feelings related to the task” (p. 423). Consistent with these points of view, in the present study, it may be the case that students’ performance (detect interference and regulate their behaviors in response to conflicts) was better when they had a higher level of self-efficacy, and these students with enhanced motivation, increased attention, and higher effortful brain activation tended to view the execution of tasks as under their control. Lewis et al. (2006)’s study focused on children of 5–16 years of age and examined the effects of negative emotion on mechanisms of response inhibition, whereas in the present study, undergraduate and graduate students engaged in the experiments and inhibition mechanisms in relation to self-efficacy were examined. Previous studies mainly focused on children (e.g., Lewis et al., 2006; Farbiash and Berger, 2015), and few of them explored the effects of cognitive factors on college students’ inhibition mechanisms from the perspective of cognitive neurology. This study provides new insights for related experimental research or theory development. Additionally, this finding is consistent with the study of Enriquez-Geppert et al. (2010), which suggested an association of the N2 with conflict-related effects with less frequently occurring trial types. In this study, No-Go is the less frequently occurring trial type, and Go stimuli were presented for most of the trials (80%). Frequent responses are prepotent, eventually leading to conflicts at the response representation level when infrequent responses have to be made (Braver et al., 2001; Jones et al., 2002; Nieuwenhuis et al., 2003).

In contrast with previous studies, this study found there was no significant difference in No-Go P3 amplitudes between the high self-efficacy group and the low self-efficacy group. The P3 component has been used to ensure that stimulus analyses are appropriately linked with the correct behavioral actions in the monitoring processes; inhibition of the action operation is associated with the No-Go P3 (Verleger et al., 2005; Smith et al., 2008). The finding of this study indicates that conflict inhibition is not modulated by self-efficacy. It contradicts the findings of Wang (2018). Wang (2018) investigated the brain inhibitory effect of self-efficacy of college students to English vocabulary stimuli and found larger No-Go P3 amplitudes in the high self-efficacy group compared with the low self-efficacy group. A possible explanation is that Wang’s study selected English biological and non-biological vocabulary as stimuli in the Go/No-Go tasks, and it is more difficult for students to identify these academic English words than the pictures used in the present study. The stimuli we used required less attention and the participants could easily withhold a dominant response to resist distraction. P3 is consciousness-dependent that is sensitive to cognitive demands during task processing such as task difficulty and the subjective probability of task stimuli or conditions (Kok, 2001; Hillman, 2004; Polich and Criado, 2006; Polich, 2007). P3 amplitude shows changes in the neural representation of the stimulus environment and reflects the allocation of task-relevant attentional control, with larger P3 amplitudes associated with the greater attentional allocation (Polich and Heine, 1996; Themanson and Rosen, 2014). Studies on attentional control implicate a network of brain regions, including the dorsolateral prefrontal cortex, parietal, and cingulate cortices (Bunge et al., 2002; Peterson et al., 2002; Durston et al., 2003; Weiss et al., 2003; Kerns et al., 2004; Langenecker et al., 2004). It is likely that due to low task difficulties for Go trials and generally reduced attention for No-Go trials, participants in the current study did not allocate much attentional control. Also, studies using different cues to induce varying levels of response preparation consistently found larger P3 amplitudes when inhibition is made more demanding (e.g., Bruin et al., 2001, Smith et al., 2007). It is possible that the effects of self-efficacy may be less powerful due to a possible increase in efficacy or confidence of participants (especially students with low self-efficacy) in their capabilities for tasks because of task repetition. The tendency of “conflict” tasks was not so large that the temptation to stop (as it is in the Go/No-Go task) called for less attentional resources or required less interference control and fewer additional cognitive loads. It also provides support for the need of exploring the mediation effects of task difficulties in future studies. Rosen (2010) and Themanson and Rosen (2014) also found students with greater self-efficacy showed larger P3 amplitudes. But they adopted a different task—a flanker task to examine the relationships between self-efficacy and neural indices of stimulus processing, task performance, and task-relevant attentional control. Their explorations only reflected part of the inhibition-related cognitive process. The elicitation and generation of the P3 component is a constant and ongoing process, and when we compare findings of different studies, a variety of associated individual difference factors have to be considered such as age, sex, intelligence, and personality (e.g., Stelmack and Houlihan, 1994; Polich, 1996). Furthermore, our findings contribute to the understanding of the social cognitive theory that explains in detail self-efficacy as a positive influence on cognitive processes (Bandura, 1993; Bandura et al., 1996; Kim, 2009; Diseth, 2011; Yusuf, 2011). Higher-order cognitive processes include not only inhibitory control but also cognitive flexibility and the ability to plan, monitor, and carry out goal-directed actions (Schacht et al., 2009). When we discuss the modulation of self-efficacy on inhibition, various associated factors could be considered.

## Conclusion

This study analyzed neural correlates of inhibition modulated by self-efficacy. It was found that students with a higher self-efficacy level tended to have a stronger ability to detect a conflict. Conflict detection was modulated by self-efficacy, whereas conflict inhibition was not modulated by self-efficacy. The high self-efficacious students were more likely to be capable of detecting conflicts but not necessarily followed by inhibition of the action operation.

In classrooms, teachers could design meaningful or student-relevant activities to increase their self-efficacy to regulate their behaviors in response to conflicts or challenges, and thus enhance motivational aspects. Teachers also could emphasize the significance of positive reinforcement and a supportive environment, and teach students problem-solving and information processing skills to encourage students to persist longer in learning tasks and engage students actively in class work.

This study explored neural correlates of inhibition modulated by self-efficacy based on Go/No-Go task monitoring and processing, but it did not reflect the whole process of inhibition and also focused on a small sample of students. Future research could implement multi-task or more complex measures to assess inhibition modulated by self-efficacy with a larger sample size. We also need to know more about how other predictors contribute further to understanding inhibition behavior change.

To sum up, the findings of this study support previous reports that inhibition in response to a stimulus is associated with self-efficacy from the perspective of cognitive neurology, and provide evidence that inhibition, indexed by N2 amplitude, may be one mechanism through which self-efficacy improves task performance. The analysis of ERP components serves to yield a more complete picture of the cognitive mechanism underlying inhibition modulated by self-efficacy. This study provides a new perspective for studies on inhibition and self-efficacy, and it will contribute to our understanding of cognitive ability development.

## Data availability statement

The original contributions presented in this study are included in the article/supplementary material, further inquiries can be directed to the corresponding author.

## Ethics statement

The studies involving human participants were reviewed and approved by China University of Petroleum-Beijing. The patients/participants provided their written informed consent to participate in this study.

## Author contributions

The author confirms being the sole contributor of this work and has approved it for publication.

## Footnotes

1
https://sccn.ucsd.edu/eeglab/index.php

## Appendix A: Self-efficacy survey

Please first answer the following questions about yourself. Your answers will be treated in a confidential manner and only identified to the researcher for this study.

1. Sex: ___________
2. Age: ___________
3. Education level: ____

The following questions ask about your self-efficacy (your self-perceptions or beliefs of capability to learn or perform tasks in classes). Answer in terms of how well the statement describes you. This usually takes about 5 min to complete. If you have any questions, let the researcher know immediately.

Please read each statement and check the box that best describes how you feel:

1 = Not at all true of me to 9 = Very true of me.

**Table:**

	Not at all true of me 1	2	3	4	5	6	7	8	Very true of me 9
I believe I will receive excellent grades in classes.									

I’m certain I can understand the most difficult material presented for classes.									

I’m confident I can understand the basic concepts taught in classes.									

I’m confident I can understand the most complex material presented by instructors in classes.									

I’m confident I can do excellent jobs on the assignments and tests in classes.									

I expect to do well in classes.									

I’m certain I can master the skills being taught in classes.									

Considering the difficulties of classes, teachers, and my skills, I think I will do well in classes.									

## Appendix B

**APPENDIX TABLE 1:** Demographic characteristics of participants.

Self-efficacy	High (*N* = 24)	Low (*N* = 26)	Total (*N* = 50)
**Sex**			
Female	12	14	26
Male	12	12	24
**Age**			
19-21	11	11	22
22-24	14	14	28
**Education Level**			
Undergraduate	11	12	23
Graduate	13	14	27

**APPENDIX TABLE 2:** Mean accuracy for all the experimental conditions.

	Trial type	High self-efficacy	Low self-efficacy
Accuracy	No-Go	0.82	0.80
	Go	0.96	0.96
Response Times(ms)	Go	410.23	412.79

**APPENDIX TABLE 3:** Analysis of variance (ANOVA) effects for N2 amplitude.

	df	F	*p*	partial η^2^
site	2,96	6.21	0.003*	0.11
site*self-efficacy	2,96	2.26	0.11	0.05
go/nogo	1,48	4.48	0.04*	0.09
go/nogo*self-efficacy	1,48	4.76	0.03*	0.09
site*go/nogo	2,96	0.97	0.38	0.02
site*go/nogo*self-efficacy	2,96	1.61	0.21	0.03
self-efficacy	1,48	7.12	0.01*	0.13

**p* < 0.05.

**APPENDIX TABLE 4:** Analysis of variance (ANOVA) effects for P3 amplitude.

	df	F	*p*	partial η^2^
site	2,96	6.74	0.002*	0.12
site*self-efficacy	2,96	0.30	0.75	0.01
go/nogo	1,48	2.19	0.15	0.04
go/nogo*self-efficacy	1,48	3.21	0.08	0.06
site*go/nogo	2,96	18.77	0.00*	0.28
site*go/nogo*self-efficacy	2,96	1.42	0.25	0.03
self-efficacy	1,48	0.03	0.86	0.00

**p* < 0.05.
